# Microwave‐Assisted Synthesis of Ir—Ni Electrocatalysts for the Oxygen Evolution Reaction in Acidic Electrolyte

**DOI:** 10.1002/open.202500279

**Published:** 2025-07-16

**Authors:** Anna Giulia Cardone, Mattia Bartoli, Adriano Sacco, Candido Fabrizio Pirri, Marco Etzi

**Affiliations:** ^1^ Center for Sustainable Future Technologies Istituto Italiano di Tecnologia Via Livorno 60 10144 Torino Italy; ^2^ Department of Applied Science and Technology Politecnico di Torino Corso Duca degli Abruzzi 24 10129 Torino Italy

**Keywords:** iridium, microwave‐assisted synthesis, nickel, oxygen evolution reaction, water splitting

## Abstract

This study investigates a microwave‐assisted synthesis method for producing Ir—Ni bimetallic catalysts for the oxygen evolution reaction in acidic environment. Due to the high cost of iridium‐based catalysts used in the anodes of proton‐exchange membrane electrolyzers, reducing the noble metal content while maintaining high performance is crucial. In this work, materials with various Ir—Ni atomic ratios are synthesized and their impact on the catalyst microstructure, phase composition, and electrochemical performance is evaluated. The results reveal a synergistic effect between the two metals, with 60 at% Ni identified as the optimal nominal composition. This catalyst achieves an overpotential of 274 mV at 10 mA cm^−2^ and a Tafel slope of 49 mV dec^−1^ in 0.5 M H_2_SO_4_ electrolyte, outperforming commercial IrO_2_ (320 mV at 10 mA cm^−2^ and 56 mV dec^−1^). The higher activity is retained after both a 6 h chronoamperometry and an accelerated degradation test, during which Ni acts as a sacrificial component and the electrochemically surface area of the films increases. Overall, this study demonstrates the potential of microwave‐assisted synthesis, a greener and faster alternative to conventional methods, for developing low Ir‐content catalysts with enhanced performance.

## Graphical abstract

This study introduces a sustainable microwave‐assisted polyol method for rapidly synthesizing Ir—Ni catalysts with enhanced oxygen evolution activity. The strong synergy between Ir and Ni delivers higher intrinsic performance than commercial IrO_2_, offering a promising alternative to conventional synthesis routes.

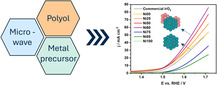

## 1 Introduction

In recent years, the decarbonization of global energy production has emerged as a critical priority in reducing greenhouse gas emissions. Renewable energy sources, such as solar and wind, are central to reaching these targets. However, their intermittent nature requires the development of efficient energy storage systems to ensure a stable and reliable supply. Water electrolysis offers a promising solution to this challenge by enabling the production of hydrogen, a versatile and clean energy vector, using only water and renewable electricity.^[^ ^1^, ^2^ ^]^ Among the different types of low‐temperature electrolyzers, proton exchange membrane (PEM) cells are very promising in terms of performance.^[^ ^3^, ^4^, ^5^ ^]^ In these electrolyzers, water is oxidized at the anode forming oxygen and protons through the oxygen evolution reaction (OER). Protons are then reduced at the cathode to produce hydrogen through the hydrogen evolution reaction (HER).^[^ ^6^, ^7^ ^]^ Despite its potential, the widespread adoption of this technology is significantly slowed down by the reliance on precious metals to efficiently drive the two half‐reactions in the acidic environment.^[^ ^3^, ^4^, ^8^ ^]^ IrO_2_ represents the benchmark catalyst for acidic OER, due to its exceptional combination of activity and stability,^[^ ^9^, ^10^, ^11^ ^]^ which makes it preferable to the more active but not sufficiently stable RuO_2_.^[^ ^12^, ^13^ ^]^ However, the high cost of Ir and the substantial loading required for commercial anodes present a major challenge for scalability. In fact, this loading often reaches up to 3 mg cm^−2^, contributing to ≈8% of the total system cost.^[^ ^1^, ^8^, ^14^, ^15^ ^]^

Consequently, several strategies have been explored to reduce or even replace the noble metals in the anodic catalyst: while the use of pure non‐noble metal catalyst is still hampered by insufficient long‐term durability,^[^ ^16^, ^17^ ^]^ co‐doping with more abundant transition metals has emerged as one of the most promising approaches. This strategy not only enhances the electrochemically active surface area (ECSA) of the doped materials but also improves their intrinsic catalytic properties.^[^ ^14^ ^]^ First‐period transition metals (e.g., Ni, Co, Cu, Cr, Mn) are commonly used as dopants to enhance the efficiency of Ir‐based catalysts.^[^ ^2^, ^15^, ^18^, ^19^, ^20^, ^21^ ^]^ For instance, Ir—Ni, Ir—Cr, and Ir—Mn oxides have demonstrated higher activity than their single‐metal Ir oxide counterparts.^[^ ^2^, ^9^, ^18^, ^22^, ^23^, ^24^ ^]^

Different synthesis methods can be used to produce bi/intermetallic nanomaterials. Among these, thermal annealing^[^ ^2^, ^25^, ^26^, ^27^ ^]^ is one of the simplest and effective methods, whereas wet chemistry^[^ ^15^, ^28^, ^29^, ^30^ ^]^ allows the production of nanocrystals with well‐controlled sizes and shapes. However, these approaches face challenges related to scalability and alignment with green chemistry principles, as they often require high temperatures, long reaction times, and the use of hazardous reagents.^[^ ^25^ ^]^ The microwave‐assisted polyol process offers an attractive alternative due to the simultaneous role of polyols as solvent and templating agents. Microwave heating further enhances the process by offering several advantages over traditional methods^[^ ^31^, ^32^ ^]^ including reduced reaction times down to a few minutes together with enhanced yields.^[^ ^22^, ^33^ ^]^ It provides uniform heating, minimizing undesired side reactions or nucleation on the vessel walls, which results in more controlled particle synthesis. Moreover, the use of closed vessels allows reactions to occur above the boiling point of the solvent, expanding the range of feasible products.^[^ ^31^, ^34^, ^35^ ^]^ These features make the microwave method a promising and cost‐effective option for catalyst production in water electrolysis.^[^ ^34^ ^]^

This study explores a microwave‐assisted synthesis method to produce Ir—Ni bimetallic particles, aiming to reduce Ir utilization in PEM electrolyzers. Ni is chosen as doping metal, as previous studies demonstrated its ability to enhance the OER activity of IrO_2_. For instance, Reier et al.^[^ ^22^ ^]^ reported improved performance for Ir—Ni mixed oxide structures, while Nong et al.^[^ ^36^ ^]^ demonstrated significant activity enhancement when replacing pure Ir with Ir—Ni core‐shell nanoparticles. We herein present a novel polyol‐assisted microwave synthesis method for the fabrication of bimetallic Ir—Ni oxygen evolution electrocatalysts. Moreover, a systematic screening of different Ir—Ni atomic ratios is performed to identify the composition that best balances catalytic performance with reduced iridium content. The synthesized catalysts are thoroughly evaluated in terms of activity and stability, while compositional and morphological analyses provide insights into the relationship between atomic composition and performance.

## 2 Results and Discussion

**Figure 1:**
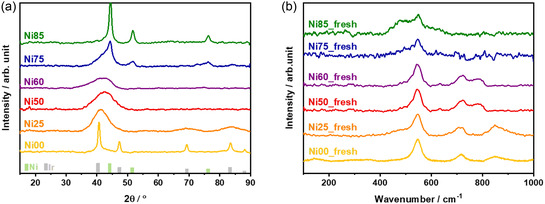
a) X‐ray diffractograms of Ir—Ni catalyst powders. Reference patterns of Ir (PDF #46‐1044) and Ni (PDF #65‐0380) are reported in the bottom part of the figure; b) Raman spectra of fresh electrodes.

Ir—Ni catalysts with different atomic ratios were synthesized and systematically characterized to identify the optimal composition and explore the factors driving the activity of the bimetallic samples. X‐ray diffraction (XRD) was used to investigate the effect of the Ir—Ni ratio on the phase composition of the catalysts (**Figure** **1**a). The resulting patterns confirm the metallic nature of the bulk samples, consistent with the reducing environment of the synthesis process, and validate microwave synthesis as an efficient method for producing mono‐ and bimetallic catalysts.^[^ ^25^, ^32^ ^]^ The diffraction patterns for the Ni00 and Ni85 samples correspond to pure iridium and nickel phases, respectively. For samples with intermediate compositions, broader and less defined peaks are observed. In the 2θ range of 40–50°, a single, broad peak combines the main reflections of iridium (40.66°) and nickel (44.35°). The asymmetry of the peak suggests the overlapping of individual metallic phases, rather than the formation of a new diffraction peak indicative of a solid solution. This interpretation is consistent with reports on nickel‐iridium alloys, where alloy formation is characterized by a single, well‐defined, and symmetric peak located approximately between the peaks of the pure phases.^[^ ^37^, ^38^ ^]^ Additionally, no peak shift is observed as iridium content increases (from Ni85 to Ni00), further supporting the conclusion that iridium is not incorporated into the nickel lattice.^[^ ^38^ ^]^

The Scherrer equation was used to determine the crystallite size of Ni00 and Ni85, as they were the only samples with well‐defined peaks suitable for this calculation. Both catalysts showed a crystallite size of ≈200 nm. This microstructural uniformity was further confirmed by field emission scanning electron microscopy (FESEM) images (Figure S1, Supporting Information), which reveal a sponge‐like microstructure for all the catalysts. However, some samples (Ni00 and Ni60) exhibit a more homogeneous porous structure, while the others display larger particles or more compact and layered arrangements. The relatively large particle size contributes to the low surface area measured by Brunauer–Emmett–Teller (BET) analysis (Figure S2, Supporting Information). All the materials were found to be mesoporous, with surface areas ranging from 1 to 10 m^2^ g^−1^ (Table S1, Supporting Information). Such low surface areas are typical for metallic powders unless specifically engineered to be more porous or structured.^[^ ^38^, ^39^, ^40^ ^]^ Moreover, the use of ethylene glycol (EG) as the only stabilizing agent in the synthesis is likely insufficient to prevent particle coalescence and agglomeration during the process.^[^ ^32^, ^41^, ^42^, ^43^ ^]^

Raman spectra of all six fresh electrodes are shown in Figure 1b. Ni00 major bands are centered at 547 and 718 cm^−1^ and they are due to the IrO_2_ phase, as reported by Pavlovic et al.^[^ ^44^ ^]^ The band centered at ≈850 cm^−1^ can be likely attributed to a defected surface or to the presence of complex species. Changes in the spectra can be observed as we change the composition of the material, due to the Ni—O bonds in different oxide species.^[^ ^45^, ^46^ ^]^ For example, Ni25 shows a minor shoulder at 475 cm^−1^ that rises in intensity by increasing the Ni amount, reaching a maximum in Ni85 sample. Since XRD diffractograms show the predominance of the metallic phase, Raman results suggest the confinement of oxide on the catalyst surface.

The elemental composition of the as‐prepared powders was qualitatively estimated by energy‐dispersive X‐ray spectroscopy (EDX) and quantitatively analyzed by inductively coupled plasma optical emission spectroscopy (ICP‐OES) (**Table** **1**). In both cases, the measured ratios deviate from the stoichiometric values of the precursors.^[^ ^18^ ^]^ For samples Ni25, Ni50, Ni60, and Ni75, higher Ir to Ni ratios are observed compared to the nominal one. This can be ascribed to the higher reduction potential of Ir, which makes it more readily reduced than Ni under the given conditions. Additionally, chloride ligands released from IrCl_4_ could strongly coordinate with Ni^2+^, forming stable complexes that may further hinder Ni reduction.^[^ ^25^ ^]^ In contrast, the Ni85 sample shows a final composition very close to the nominal one, likely due to the low Ir content in the precursor mixture, which limits its competitive role in the reduction process.

**Table 1:** EDX and ICP‐OES elemental composition analysis of Ir—Ni catalysts.

	EDX		ICP‐OES	
Sample	Ni: (Ni + Ir) [at%]	Ir: (Ni + Ir) [at%]	Ni: (Ni + Ir) [at%]	Ir: (Ni + Ir) [at%]
Ni25	12 ± 4	88 ± 4	21	79
Ni50	38 ± 5	62 ± 5	–	–
Ni60	43 ± 10	57 ± 10	51	49
Ni75	53 ± 4	47 ± 4	–	–
Ni85	90 ± 5	10 ± 5	–	–

Two samples (Ni25 and Ni60) were analyzed by ICP‐OES to precisely quantify the Ir:Ni ratio. Compared to the EDX data, the slightly higher Ni content measured by ICP‐OES for Ni25 may reflect a nonuniform distribution within the powder or indicate the presence of subsurface Ni species that are not accessible to more surface‐sensitive techniques. The Ni60 results are not significantly different considering the related uncertainties of the values. However, in both cases, the amount of Ni is still lower than the nominal one, for the reasons described above. While these deviations are critical for evaluating the optimal catalyst composition, for clarity, the samples will continue to be identified based on the nominal Ni atomic percentage used in the precursors.

The surface chemical composition of the as‐prepared Ni00, Ni25, and Ni60 samples was analyzed through X‐ray photoelectron spectroscopy (XPS). The survey spectrum (Figure S3, Supporting Information) reveals the presence of four elements, namely C, Ir, Ni, and O. The surface composition of the Ni25 and Ni60 samples determined by XPS (Table S2, Supporting Information) shows slightly lower Ni contents than those estimated by EDX. These results indicate a surface enrichment of Ir, in accordance with previous studies of doped IrO_2_.^[^ ^2^, ^47^ ^]^ The Ir 4f regions of the selected samples are shown in Figure S4 (Supporting Information). The binding energy (*E*_*b*_) of Ir 4f_7/2_ (*E*_*b*_ ≃ 61.5–61.7 eV) peaks fall between those of IrO_2_ (whose spectrum is also reported in Figure S4 (Supporting Information), *E*_*b*_ ≃ 62.5 eV)^[^ ^18^, ^21^, ^48^ ^]^ and those of metallic Ir (*E*_*b*_ ≃ 60.8 eV),^[^ ^48^ ^]^ indicating that the surface of Ir particles is partially oxidized. Moreover, when comparing the Ir 4f regions of Ni00 with those of Ni25 and Ni60, we can observe a slight shift of Ir 4f peaks toward lower binding energies in the latter two samples (0.2 eV). This result suggests that Ni addition partially modifies the chemical environment of Ir, leading to the formation of more reduced Ir particles. This is consistent with previous studies showing reduced binding energies for Ir in Ir—Ni structures compared to pure metallic nanoparticles, although energy shifts to values below the metallic reference are more commonly observed in Pt alloys than in Ir alloys.^[^ ^9^, ^18^, ^49^ ^]^

**Figure 2:**
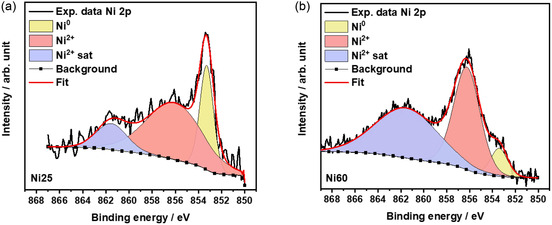
XPS fitting of the Ni 2p_3/2_ region of a) Ni25 and b) Ni60.

Ni 2p_3/2_ region of Ni25 and Ni60 samples (**Figure** **2**a,b) shows three distinct contributions that reveal the presence of Ni oxide species on the material surface. These contributions at ≈853.3, 856.2, and 861.7 eV correspond to Ni^0^, Ni^2+^, and Ni^2+^ satellite species, respectively.^[^ ^38^, ^50^, ^51^ ^]^ Notably, Ni60 exhibits a higher concentration of Ni^2+^ species compared to Ni25, indicating that the Ni‐rich sample is initially more oxidized. Combining the analysis of both Ir and Ni XPS regions, we can observe that increasing the Ni content leads to higher contents of reduced Ir and oxidized Ni species in the sample (Ni60), suggesting the potential occurrence of a galvanic reaction between Ir and Ni.

O 1s spectra (Figure S5, Supporting Information) show three oxygen species at ≈529.8, 531.8, and 533.0 eV. The lower‐energy peak corresponds to lattice oxygen, while the others are attributed to hydroxyl groups and adsorbed water, respectively.^[^ ^21^, ^22^ ^]^ This analysis confirms the presence of oxidized species and reveals the existence of surface hydroxyl groups, which may contribute to the enhanced catalytic activity of the Ir—Ni systems.^[^ ^22^, ^52^ ^]^

**Figure 3:**
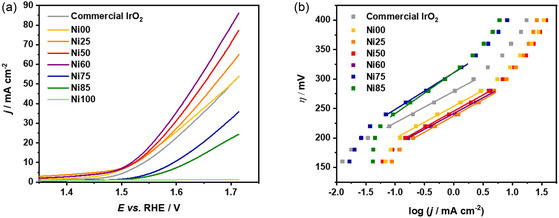
a) LSV and b) Tafel plot recorded in 0.5 M H_2_SO_4_.

The OER activity of Ir—Ni catalysts was assessed in 0.5 M H_2_SO_4_ through linear sweep voltammetry (LSV) and Tafel plots. **Figure** **3**a reports the OER voltammograms obtained for the different catalysts. Ni00 exhibits an activity comparable to that of a commercial IrO_2_ catalyst tested as a benchmark. When the Ni content increases, i.e., for samples Ni25, Ni50, and Ni60, we can observe a pronounced cathodic shift and higher current densities in the whole potential range, indicating improved catalytic activity of these mixed Ir—Ni samples compared to pure Ir. However, at higher Ni concentrations (Ni75 and Ni85), we can observe a decay in performance, as shown by the lower current densities reached even at high potential. These results are likely due to the inactivity of pure Ni (Ni100), which shows the lowest performance in the LSV. A key metric for comparing the catalytic efficiency of different materials is the *η*_10_, which is the overpotential required to reach a geometric current density of 10 mA cm^−2^.^[^ ^53^ ^]^ As summarized in **Table** **2**, *η*_10_ decreases with increasing Ni content from Ni00 to Ni60, reaching a minimum of 274 mV at a nominal Ir—Ni ratio of 40:60. This value is almost 50 mV lower than the *η*_10_ of the commercial IrO_2_ tested as reference material. Such overpotential, lying within the 200–300 mV range, aligns well with the benchmarks for excellent OER catalysts in acidic environments.^[^ ^54^, ^55^ ^]^ For Ni75 and Ni85 samples, the *η*_10_ is higher, further demonstrating the low activity of these Ni‐rich samples.

**Table 2:** Summary of overpotential and Tafel slope values of different catalysts evaluated in 0.5 M H_2_SO_4_.

Sample	*η*_10_ [mV]	Tafel slope [mV dec^−1^]
Commercial IrO_2_	320 ± 9	56 ± 3
Ni00	289 ± 14	56 ± 3
Ni25	281 ± 9	52 ± 1
Ni50	282 ± 13	51 ± 5
Ni60	274 ± 14	49 ± 7
Ni75	380 ± 12	62 ± 5
Ni85	401 ± 19	68 ± 4

The Tafel plots obtained by step chronoamperometry (step CA) for all the samples are shown in Figure 3b. The slope of the linear region of these plots, known as the Tafel slope, is used to evaluate the activity of the catalysts. Indeed, a lower slope reflects improved reaction kinetics, indicating that small increases in potential lead to substantial increases in current density. The Tafel slope of the Ni00 sample (56 mV dec^−1^) matches that of the commercial IrO_2_ (56 mV dec^−1^), once again demonstrating comparable performance with the benchmark catalyst. This slope value is consistent with previously reported values for crystalline iridium and IrO_2_.^[^ ^2^, ^56^ ^]^ Ni25 (52 mV dec^−1^), Ni50 (51 mV dec^−1^), and Ni60 (49 mV dec^−1^) outperform both the metallic Ir and the commercial IrO_2_, suggesting that Ni addition can enhance the reaction kinetics.^[^ ^2^, ^56^ ^]^ In contrast, Ni75 and Ni85 samples exhibit higher slopes, indicating poorer performance due to insufficient Ir content.

The synergistic effect responsible for the enhanced activity, enabling the bimetallic catalyst to outperform both pure Ir and Ni, is well‐documented in the literature. Yan et al. reported that the introduction of transition metals into Ir matrices can fine tune the electronic properties of active sites, reducing the adsorption energy of key intermediates.^[^ ^38^ ^]^ Wang et al. attributed the optimization of binding energies for critical reaction intermediates (O*, OH*, and OOH*) to lattice contraction and a shift in the d‐band center of Ir within the bimetallic structure.^[^ ^57^ ^]^ This lattice contraction is also evident in our samples when comparing the XRD patterns of Ni00 and Ni25. Specifically, the Ir‐related peak at ≈41° shifts to a higher diffraction angle after the addition of Ni, indicating a reduction in the interplanar spacing of the Ir lattice. Moreover, other researchers attributed the improved performance of Ir—Ni catalysts in acidic environments to the formation of surface OH groups and electronic defects caused by Ni dissolution.^[^ ^22^, ^52^ ^]^ Indeed, they demonstrated that as Ni atoms dissolve from the oxidized NiO surface, destabilized oxygen atoms interact with protons to form surface hydroxide groups. These groups are hypothesized to act as critical intermediates in the OER mechanism, enhancing the kinetics of the reaction.^[^ ^22^, ^52^ ^]^

Overall, we can infer that the predominance of Ir is essential for achieving high catalytic performance, as evidenced by the activity decay at higher Ni compositions. Moreover, as follows from EDX results, we can conclude that the samples containing between 15 and 40 Ni at% are the catalysts with the highest intrinsic catalytic performance, likely due to an optimal alloy ratio that enhances the formation of effective reactive centers.^[^ ^38^ ^]^

**Figure 4:**
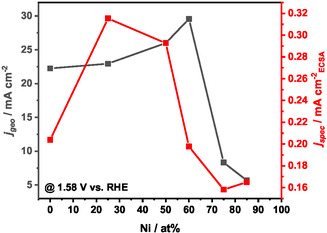
Geometric versus specific current density of Ir—Ni catalysts evaluated in 0.5 M H_2_SO_4_.

The ECSA values reported in **Table** **3** were calculated as the ratio of the double‐layer capacitance obtained by electrochemical impedance spectroscopy (EIS) (data fitting in Figure S6, Supporting Information) with the specific capacitance. These values were used to normalize the current densities recorded at *η *= 350 mV during the LSV measurements, resulting in the so‐called *j*_spec_. This parameter represents a measure of the intrinsic activity of the catalysts, as it reflects the specific current density per unit of active surface area. As shown in **Figure** **4**, these data were then compared to the current densities normalized on the geometric area of the films (*j*_geo_) collected from LSV measurements.

**Table 3:** Electrochemically active surface area values measured by EIS.

Sample	ECSA [cm^2^]
Commercial IrO_2_	27
Ni00	54
Ni25	36
Ni50	44
Ni60	74
Ni75	27
Ni85	17

Ni00 and Ni60 show the largest active surface areas among all the samples (54 and 74 cm^2^, respectively). On the other hand, the Ni‐rich samples exhibit the lowest ECSA values, which partially explains their reduced catalytic activity. Despite these differences, the ECSA values across all catalysts remain within the same order of magnitude.

*J*_geo_ at *η* = 350 mV exhibits a strong dependence on the Ni content. Specifically, it increases progressively from 22 mA cm^−2^ for Ni00, to a maximum of 30 mA cm^−2^ for Ni60. As the Ni content continues to rise, *j*_geo_ drops significantly partly due to the lower ECSA values. A similar trend is observed for *j*_spec_, which initially increases with the Ni content, peaking at values of 0.32 and 0.30 mA cm^−2^_ECSA_ for the Ni25 and Ni50 samples, respectively. However, it decreases at higher Ni contents (e.g., 0.20 mA cm^−2^_ECSA_ for Ni60 sample). This divergence from *j*_geo_ suggests that the overall performance is maximized at a nominal Ir—Ni ratio of 40–60 at% due to high exposed active area, but the intrinsic catalytic activity of the bimetallic material decreases before reaching this composition. As the Ni content further increases, *j*_spec_ continues to decay, confirming the inherently lower intrinsic catalytic activity of these catalysts. Moreover, a comparative analysis of the ECSA and *j*_spec_ values for Ni00 (54 cm^2^, 0.20 mA cm^−2^_ECSA_), Ni25 (36 cm^2^, 0.32 mA cm^−2^_ECSA_), and Ni50 (44 cm^2^, 0.30 mA cm^−2^_ECSA_) highlights the dual effect of Ni incorporation. Specifically, the addition of Ni not only modifies the catalyst morphology, thereby influencing the ECSA, but also enhances the intrinsic catalytic activity of the material.

We also compared our electrocatalytic activity data with those previously reported in the literature. Unfortunately, a fair comparison of performance across materials reported is hampered by the lack of standardized testing protocols: OER catalysts are deposited onto various substrates with different loadings, and their electrocatalytic activity is evaluated under diverse conditions, such as pH and electrolyte concentration. This lack of standard testing conditions makes it challenging to directly compare results across studies. Nevertheless, **Table** **4** summarizes the performance of several bimetallic catalysts for OER in acidic environments, based on two of the most used activity metrics.

**Table 4:** Literature review on bimetallic materials as OER electrocatalysts (Cu_1.11_Ir NCs: single‐crystalline Cu—Ir polyhedral nanocages. Ni_2.53_Ir NCs: polycrystalline Ni—Ir nanocages. Ir_1−*x*_ Ni_*x*_O_2−*y*_: Ir–Ni Composite Oxide (0 ≤ × ≤ 0.5). Ir‐based NFs: IrNi alloy nanoflowers. Ir_6_Ag_9_ NTs: hollow Ir—Ag nanotubes. IrCrOx: electrochemically oxidized IrCr thin film. IrO_2_@RuO_2_: core‐shell‐like bimetallic mixed oxide. Pt—Ir NOs: nano‐octahedra Pt‐Ir alloy. Rh_22_Ir_78_/VXC: Rh—Ir alloy nanoparticles deposited on Vulcan XC‐72R carbon black).

Electrocatalyst	Electrolyte	*η*_10_ [mV]	Tafel slope [mV dec^−1^]	References
Cu_1.11_Ir NCs	0.05 M H_2_SO_4_	286	44	[59]
Ni_2.53_Ir NCs	0.05 M H_2_SO_4_	302	47	[57]
Ir_1−*x*_ Ni_*x*_O_2−*y*_	0.5 M H_2_SO_4_	330	–	[58]
Ir‐based NFs	0.1 M HClO_4_	293	47	[37]
Ir_6_Ag_9_ NTs	0.5 M H_2_SO_4_	285	88	[60]
IrCrOx	0.5 M H_2_SO_4_	390	59	[9]
IrO_2_@RuO_2_	0.5 M H_2_SO_4_	270	58	[61]
Pt‐Ir NOs	0.5 M H_2_SO_4_	300	79	[62]
Rh_22_Ir_78_/VXC	0.5 M H_2_SO_4_	292	–	[63]
Ni60	0.5 M H_2_SO_4_	274	49	This work

Ir—Ni catalysts are widely studied for their promising activity, and the bimetallic catalyst presented in this work demonstrates performance that slightly exceeds that of previously reported systems.^[^ ^37^, ^57^, ^58^ ^]^ Cu is another widely investigated transition metal due to its low cost and excellent conductivity. However, its application in acidic environments remains challenging because of its poor chemical stability.^[^ ^59^ ^]^ Similarly, Ni acts as a sacrificial component under acidic conditions, but the formation of nickel oxide and hydroxide layers after exposure to H_2_SO_4_ electrolyte has been shown to enhance catalytic activity.^[^ ^22^, ^52^ ^]^ An alternative approach is proposed by Zhu et al.,^[^ ^60^ ^]^ who investigated the use of Ag as a doping element. While effective, this method relies on a wet chemistry synthesis process that is less environmentally sustainable and more costly and time‐consuming. Other transition metals have shown exceptional catalytic properties. For instance, IrO_2_–RuO_2_ systems achieve a low overpotential of 270 mV at 10 mA cm^−2^.^[^ ^61^ ^]^ However, the reliance on platinum group metals (PGMs), including Ru, Pt, and Rh, remains a significant drawback due to their scarcity, high cost, and environmental concerns,^[^ ^61^, ^62^, ^63^ ^]^ as previously discussed. In this context, the Ir—Ni catalyst proposed in this study emerges as a promising alternative, combining simple synthesis, earth‐abundant materials, and high performance under acidic conditions, offering a sustainable and cost‐effective solution.

**Figure 5:**
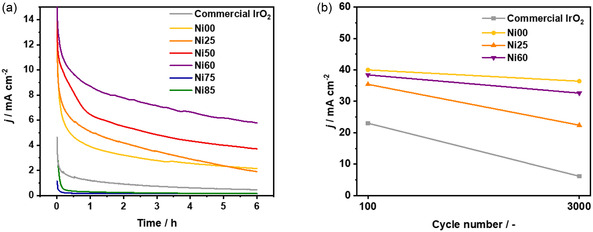
a) 6 h chronoamperometry at 1.53 V versus RHE in 0.5 M H_2_SO_4_. b) Measured current densities at 1.6 V versus RHE in 0.5 M H_2_SO_4_ during ADT.

Apart from the activity, the stability of the catalyst is another important parameter to take into account. We evaluated the stability of our catalysts through a 6 h chronoamperometry at 1.53 V (**Figure** **5**a). Ni00 sample demonstrates a stability behavior partially resembling that of commercial IrO_2_: after a higher initial decrease in current density, the current density decay becomes comparable to that of the commercial catalyst, indicating similar long‐term stability. In contrast, the CA curves for Ni25, Ni50, and Ni60 samples display significantly steeper slopes, reflecting a faster degradation during operation. The degradation rate was quantified as the ratio between the difference in final and initial current densities and the total duration of the test (**Table** **5**). Compared to the commercial IrO_2_, all the synthesized metallic catalysts exhibit higher degradation rates. This can be attributed to their metallic nature, as highlighted by previous studies. Strickler et al.^[^ ^9^ ^]^ and Jovanovič et al.^[^ ^44^ ^]^ demonstrated that the metallic nature of bimetallic catalysts results in higher Ir dissolution rates compared to preoxidized Ir. Moreover, the dissolution of Ir can be further accelerated in such systems due to the formation of less stable Ir sites during the leaching of non‐noble metals. Özer et al.^[^ ^52^ ^]^ also found that Ni incorporation significantly enhances the electrocatalytic activity of iridium, while adversely affecting stability. However, even after 6 h of operation, the bimetallic catalysts presented here retain higher current densities than commercial IrO_2_.^[^ ^18^, ^22^ ^]^

**Table 5:** Degradation rate of the catalysts measured as the decay in the current density during a 6 h CA (1.53 V versus RHE in 0.5 M H_2_SO_4_) stability test and a 3000‐cycle ADT (0.05 V and 1.6 V versus RHE in 0.5 M H_2_SO_4_).

Sample	CA_Degradation rate [mA cm^−2^ h^−1^]	ADT_Degradation rate [mA cm^−2^ cycle^−1^]
Commercial IrO_2_	0.51	0.58
Ni00	0.76	0.12
Ni25	0.97	0.44
Ni50	0.85	–
Ni60	0.81	0.20
Ni75	0.34	–
Ni85	0.25	–

Although the degradation rates for Ni75 and Ni85 are low, the extremely low initial activity and current density in the CA curves should be considered, making the calculation inadequate for a reasonable comparison of stability.

To enable a rapid evaluation of long‐term electrocatalyst stability, the 6‐h CA test was integrated with an accelerated degradation test (ADT), as shown in Figure 5b. This test enables to mimic the behavior of the catalyst under continuous start‐and‐stop conditions (3000 cycles).

The current densities measured at 1.6 V in the 100th and 3000th cycles were compared. First, the current densities of all the samples are higher than that of commercial IrO_2_ in the 100th cycle. At the end of the test, the trend of decrease in current densities is similar. Ni00 is the most active sample followed by Ni60.

The decrease in current density per cycle was evaluated and reported in Table 5. In contrast to the short‐term CA results, Ni‐containing samples exhibit long‐term stability comparable to that of commercial IrO_2_, with even lower degradation rate (Table 5). This behavior is likely associated with the initial dissolution of Ni species, which accounts for the reduced stability observed in the early stages.

The electrolytes after ADT were collected and successively analyzed by ICP‐OES to determine the amount of dissolved Ir and Ni species (**Table** **6**).

**Table 6:** Amount of Ir and Ni in the as‐deposited electrode and amount of Ir and Ni dissolved after 3000 cycles of ADT.

	Dissolved Ir [μg cm^−2^]	Dissolved Ni [μg cm^−2^]
Ni00	57	–
Ni25	35	7
Ni60	46	34

The amount of dissolved Ir in samples Ni25 and Ni60 is lower than for Ni00. For these samples, we found that Ni completely leached during the ADT. In bimetallic systems, Ir dissolution is typically more pronounced than in pure Ir films. This is attributed to the formation of less stable Ir sites following the leaching of the non‐noble metal, as previously reported by Alaina et al.^[^ ^9^ ^]^ However, Ir dissolution in our samples is similar, indicating that the stability of Ir is not hampered by the initial presence of Ni and that Ni leaching could also lower Ir dissolution. The complete Ni dissolution may result from the insufficient development of a stable and protective Ir‐enriched surface.^[^ ^9^ ^]^

The impact of the electrochemical test on the catalysts was further evaluated by CV, EIS, FESEM, EDX, XRD, and Raman analyses of the used electrodes after the 6 h CA test.

**Figure 6:**
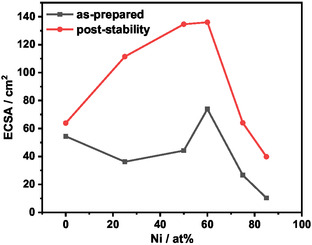
ECSA value of as‐prepared versus spent electrode after 6 h CA at 1.53 V versus RHE in 0.5 M H_2_SO_4_.

EDX measurements show that Ni is detected in trace amounts after the 6 h stability test (Table S3, Supporting Information). As suggested in the literature, Ni could act as a sacrificial component, and it is the first to be leached out.^[^ ^22^ ^]^ As a result, in the Ni‐depleted region, hydroxide groups could replace the oxidized surface layer, explaining the high activity of the bimetallic.^[^ ^18^, ^22^ ^]^ The CV profiles (Figure S7, Supporting Information) show significant changes, becoming very similar to that of IrO_2_ (Figure S8, Supporting Information).^[^ ^22^ ^]^ Specifically, the main peak at ≈1 V (Ir(III)/Ir(IV) redox) which was previously visible only for Ni00, Ni25, and Ni50 samples becomes prominent for all the catalyst compositions. This further demonstrates the dissolution of Ni and the oxidation of Ir during the electrolysis.^[^ ^22^, ^64^ ^]^ These results are in agreement with the Raman spectra of the spent electrodes (Figure S9, Supporting Information): they present an envelopment of bands centered at 540 cm^−1^ due to the superimposition of both Ir—O and Ni—O modes. However, the contribution of the NiO mode appears significantly reduced compared to the fresh samples, in line with the complete dissolution of Ni during the ADT shown by ICP‐OES. The ECSA of the working electrode increases after the test (**Figure** **6**) leading to the formation of a more cracked film and a finer microstructure.^[^ ^18^, ^22^ ^]^ Among the samples, Ni00 exhibits the smallest ECSA increment, consistent with the greater stability of noble metals. In contrast, Ni75 and Ni85 show only a limited increase in surface area, likely caused by partial detachment of the catalyst film and extensive Ni dissolution. The crystal structure of the spent electrodes was investigated by XRD and grazing incidence XRD (GI‐XRD); however, only reflections from the glassy carbon substrate were detected (Figure S10 and S11, Supporting Information). Microstructural comparisons by FESEM (Figure S12, Supporting Information) indicate that the overall film remains homogeneous after testing. Ni00 exhibits the highest structural stability during the test, followed by Ni25 and Ni50. In contrast, starting from Ni60, progressive structural degradation is observed, including cracking and localized detachment as visible in Ni85. In addition, particle size refinement is partially visible in all the samples, supporting the increase in the total exposed area shown by ECSA measurement.

## 3 Conclusions

This study introduces a microwave‐assisted synthesis method for preparing Ir—Ni bimetallic catalysts for acidic water oxidation. A range of Ir—Ni atomic ratios was evaluated to investigate the effects of Ni incorporation on the microstructure, phase composition, electrocatalytic activity, and stability of Ir‐based electrodes for the OER. Based on the combined analysis of XRD, Raman, and XPS results, we propose that the catalyst particles consist of a metallic core surrounded by a surface‐confined oxide layer. Moreover, the XRD patterns reveal the characteristic reflections of metallic Ir and Ni, indicating the presence of separate crystalline phases; meanwhile, XPS and Raman spectroscopy detect signals corresponding to IrO_2_ and Ni—O bonds, without evidence of alloy formation. These observations suggest that Ni segregates forming distinct particles on the Ir particle surface.

A synergistic effect between Ir and Ni resulted in catalysts with enhanced intrinsic performance compared to the pure metals and the commercial IrO_2_ benchmark. Among the tested compositions, the Ni60 catalyst exhibits the highest activity, achieving an overpotential of 274 mV at 10 mA cm^−2^ and a Tafel slope of 49 mV dec^−1^. These values place Ni60 within the range typically associated with excellent OER catalysts in acidic environments. The catalyst also exhibits good stability, with a current density after a 6 h‐chronoamperometry significantly exceeding that of commercial IrO_2_, despite a higher degradation rate attributed to its metallic nature. However, at longer timescales, Ni‐containing samples display stability comparable to IrO_2_, likely as a result of the stabilization following the initial leaching of Ni species. Ir leaching was found even lower for samples Ni25 and Ni60 than for Ni00, indicating that Ni leaching partly protected Ir from dissolution. The current densities at the end of an ADT were still higher for Ni00 and Ni60 compared to commercial IrO_2_, indicating the stability of the microwave‐synthesized catalysts. Poststability analyses using CV, EDX, ECSA, and ICP‐OES reveal that Ni leached out during the reaction. This process creates Ni‐depleted regions with finer microstructure and larger electrochemically active surface area. Overall, this study demonstrates the feasibility of employing a simple and scalable synthesis method for developing low Ir content catalysts with optimized performance.

## 4 Experimental Section

4.1

4.1.1

##### Materials

Iridium (IV) chloride (99.95%, Thermofisher), nickel (II) acetate tetrahydrate (98%, Sigma–Aldrich), EG (>99.5%, Supelco), and sodium hydroxide (>98%, Sigma–Aldrich) were used as received, without further purification. Sulfuric acid (95.0–97.0%, Sigma–Aldrich) was diluted using deionized water (resistivity 18.2 MΩ·cm, Milli‐Q purification system) to prepare an electrolyte with a 0.5 M concentration. Nafion 117 (5 wt% solution) was supplied by Sigma–Aldrich. Iridium (IV) oxide (99.99% Premion, Thermofisher) was used as a benchmark catalyst.

##### Methods: Synthesis

Ir—Ni catalysts were synthesized using a microwave‐assisted polyol method, following established protocols in the literature.^[^ ^25^, ^65^ ^]^ Iridium (IV) chloride and nickel (II) acetate tetrahydrate served as metal precursors. EG was utilized as both solvent and reducing agent. Sodium hydroxide was added to accelerate the reduction kinetics, as the inclusion of a base facilitates the deprotonation of the alcohol groups in EG, promoting the formation of alkoxide monoanions; these species are more reactive and exhibit a higher affinity for coordination with metal ions.^[^ ^25^, ^34^ ^]^

The two precursors were dissolved in EG in seven different Ir—Ni atomic ratios (100:0, 75:25, 50:50, 40:60, 25:75, 15:85, and 0:100) but keeping a constant total concentration of 10 mg mL^−1^. NaOH was added up to a concentration of 4 mg mL^−1^. The resulting solution was stirred until a uniform color was observed and then transferred into an Anton Paar Monowave 200 microwave reactor. Monometallic Ir (Ni00) catalyst was synthesized by heating the solution to 160 °C with a ramp‐up time of 1 min, followed by a 5 min hold. Ni‐containing samples required higher synthesis temperatures, as the reduction of nickel acetate in EG has been shown to occur only at temperatures between 200 and 250 °C, depending on the presence of additional promoting agents.^[^ ^25^ ^]^ Therefore, for bimetallic Ir—Ni and pure Ni (Ni100) samples, the solution was heated to 250 °C over 3 min and maintained at this temperature for 25 min. Rapid cooling to 60 °C was followed in both cases, with stirring maintained at 1200 rpm throughout the process. Ir nanoparticles were precipitated from EG using 1 M HCl, while Ir—Ni nanoparticles were collected with 0.5 M HCl to prevent Ni degradation. This precipitation step is critical when using EG as a solvent, as demonstrated in previous studies.^[^ ^41^, ^65^, ^66^ ^]^ After precipitation, the powders were washed three times with 0.1 M HCl and collected via centrifugation at 4500 rpm for 5 min. Finally, the samples were dried under vacuum at 40 °C overnight. The samples are labeled as Ni*xx*, where *xx* represents the atomic percentage of Ni precursor.

##### Methods: Electrode Preparation

The synthesized powders were used to prepare an ink, subsequently deposited onto a glassy carbon electrode. The deposition area (0.5 cm^2^) was defined using Teflon tape and the back of the electrode was covered by Kapton tape. Before deposition, the glassy carbon substrates were polished with a 0.05 μm alumina suspension on an alumina polishing pad, followed by rinsing with distilled water. The following method adapted from the literature^[^ ^21^ ^]^ was used to prepare the ink: a mixture containing 2 mg of catalyst, 20 μL of Nafion, 780 μL of Milli‐Q water, and 200 μL of isopropanol was sonicated in an ultrasonic bath until a homogeneous suspension was achieved. In total, 75 μL of this ink was drop‐casted onto the glassy carbon electrode in three increments of 25 μL, with 10 min of drying at 60 °C between each step. The final catalyst loading on the electrode was 300 μg cm^−2^. The same ink and deposition steps were used for the preparation of a commercial IrO_2_ catalyst used as a benchmark. To assess the reproducibility of the results and to calculate the standard deviation, at least two electrodes for each catalyst composition were tested in each electrochemical analysis.

##### Methods: Electrochemical Characterization

Electrochemical measurements were performed at room temperature using a three‐electrode setup connected to an Autolab PG‐STAT30 potentiostat (Metrohm Autolab B.V.). The catalyst‐covered glassy carbon electrode was used as working electrode, while a platinum coil and an Ag/AgCl electrode (RE‐1B, ALS) served as counter and reference electrodes, respectively. The electrolyte for all the experiments consisted of 40 mL of 0.5 M H_2_SO_4_. Reported potentials were corrected for 85% of the *iR*‐drop and referenced to the reversible hydrogen electrode (*V*_RHE_) according to the following equation

(1) $$V_{\text{RHE}}=V_{\text{meas}\text{.}}+0.195+0.059\times \text{pH}$$

where *V*_meas._ is the measured potential by the reference electrode, 0.195 V is the potential of the Ag/AgCl reference electrode versus SHE, and pH was determined to be 0.3.

Cyclic voltammetry (CV) was performed in a potential range of 0.0–1.4 V versus RHE at a scan rate of 50 mV s^−1^ to identify the redox window and assess the capacitive current response.^[^ ^67^ ^]^ After stability testing, CV measurements were repeated to evaluate potential changes in the catalyst composition.

Catalytic activity was evaluated via LSV, recorded in the anodic direction at a scan rate of 10 mV s^−1^ within a 0.7–1.7 V versus RHE potential window. Tafel plots were derived from step chronoamperometry (CA) experiments, where the applied potential was increased by 20 mV every 60 s within a range of 1.3–1.8 V versus RHE. This hold time was sufficient to minimize capacitive current while preventing significant catalyst degradation. The steady‐state current density at each potential was used to construct the Tafel plots, and the slope of their linear region was analyzed to assess catalytic performance.

Catalyst stability was evaluated via chronoamperometry at a constant potential of 1.53 V versus RHE for 6 h. An ADT based on the protocol developed by Spöri et al.^[^ ^68^ ^]^ was also performed for a rapid and reproducible assessment of catalyst long‐term stability. This transient ADT consists of 3000 potential square‐wave cycles between 0.05 and 1.6 V versus RHE, with 3 s holding time per step. This approach effectively simulates the on/off operating conditions of PEM electrolyzers, yielding degradation trends that are quantitatively comparable to those obtained from conventional chronoamperometry tests conducted over significantly longer durations.

The electrolytes were collected and successively analyzed by ICP‐OES using a ThermoFisher iCAP 7600 DUO Thermospectrometer to determine the amount of dissolved Ir and Ni species.

The degradation rate of the catalysts during the 6 h CA was calculated from the current density decrease between *t* = 10 min (after capacitive effects had decayed) and *t* = 6 h, corresponding to an effective duration of 5 h 50 min. For the ADT, the degradation rate was defined as the current density loss per cycle, calculated between cycle 100 and cycle 3000.

The ECSA was determined using EIS. The double‐layer capacitance (*C*_*dl*_) of the electrodes was calculated and used to estimate the ECSA using the equation

(2) $$ECSA=\frac{C_{dl}}{C_{s}}$$

where *C*_*s*_ represents the specific capacitance, assumed to be 0.035 mF cm^−2^ for metal electrodes in acidic media.^[^ ^69^, ^70^ ^]^ EIS measurements were performed in static electrolyte conditions at a fixed potential (0.41 V versus RHE) corresponding to the capacitive region observed in CV. A sinusoidal signal with an amplitude of 10 mV was applied across a frequency range from 0.1 to 10^5^ Hz. Data fitting to the *R*_*s*_(*R*_*ct*_ *C*_*dl*_) equivalent circuit model was performed using Nova 2.1.7 software, where *R*_*s*_ represents the electrolyte resistance, *R*_*ct*_ the charge transfer resistance, and *C*_*dl*_ the double‐layer capacitance of the electrodes. *C*_*dl*_ measured by EIS was selected as the preferred method for ECSA determination since the CV curves of our fresh samples did not exhibit evident redox peaks in the potential window of 0.4–1.4 V versus RHE, making it impractical to estimate the anodic charge (q*) and therefore ECSA through this method.^[^ ^71^, ^72^ ^]^

##### Methods: Materials Characterization

Powder XRD analysis was performed using a PANalytical X’Pert PRO diffractometer equipped with a Cu Kα radiation source. The diffraction patterns were recorded over a 2θ range of 15–90° with a step size of 0.026°. The Scherrer equation (Equation (3)) and the HighScore Analysis Software were used to calculate the crystallite size (*D*), according to the following equation.

(3) $$D=\frac{\lambda k}{\beta cos\theta }$$

where *λ* is the X‐ray wavelength, *k* is the shape factor (0.9), *β* is the Full Width Half Maximum (FWHM, and θ is the Bragg's diffraction angle.

Grazing incidence XRD (GI‐XRD) was used for thin‐film analysis on the spent electrode. The diffraction pattern was recorded over a 2θ range of 30°–55°, with a step size of 0.026° and an acquisition time of 12 s per step, using a fixed incidence angle of 1°.

FESEM was conducted with a ZEISS Supra microscope at an accelerating voltage of 5 kV to analyze particle morphology and the uniformity of the deposited layer. EDX at 15 kV was used to evaluate elemental composition. Both techniques were applied before and after the stability test to assess structural and compositional changes.

The elemental composition of Ni00, Ni25, and Ni60 samples was further determined by ICP‐OES using a ThermoFisher iCAP 7600 DUO Thermospectrometer. To perform the analysis, the powders were first digested in 1 mL of Aqua Regia at room temperature and brought to a final volume of 10 mL with MilliQ water.

XPS measurements of selected catalysts were carried out on a PHI 5000 Versaprobe spectrometer equipped with a monochromatic Al Kα (1486.6 eV) X‐ray source. An electron gun and an Ar ion gun were used as charge compensation system. The spot size was 100 μm and the pass energy was set at 187.85 and 23.5 eV for survey and high‐resolution scans, respectively. Binding energy calibration was applied by setting the position of the C 1s sp^3^ peak at 284.8 eV. The spectra were processed using CasaXPS software (v2.3.23, Casa Software Ltd).

The specific surface area, pore volume, and average pore size of the powdered samples were determined using BET analysis. Measurements were conducted with a Micromeritics ASAP 2020 Plus instrument. Sample degassing was carried out with a heating ramp of 10 °C min^−1^, a holding temperature of 180 °C, and a holding time of 180 min. The analysis was performed using the standard program for a silica‐alumina reference material, with N_2_ as adsorbing gas.

Raman spectroscopy was performed using a LabRAM Soleil multimodal Raman spectrometer with an exciting laser of 785 nm at a temperature of 298 K in the range 100–1000 cm^−1^.

## Conflict of Interest

The authors declare no conflict of interest.

## Author Contributions

**Anna Giulia Cardone**: conceptualization (supporting); data curation (lead); formal analysis (lead); investigation (lead); methodology (equal); validation (lead); visualization (lead); writing—original draft (lead); writing—review and editing (equal). **Mattia Bartoli**: data curation (supporting); formal analysis (supporting); investigation (supporting); methodology (supporting); visualization (supporting); writing—review and editing (supporting). **Adriano Sacco**: methodology (supporting); supervision (supporting); writing—review and editing (supporting). **Candido Fabrizio Pirri**: funding acquisition (lead); resources (lead). **Marco Etzi**: conceptualization (lead); data curation (supporting); formal analysis (supporting); investigation (supporting); methodology (equal); project administration (lead); supervision (lead); validation (supporting); visualization (supporting); writing—original draft (supporting); writing—review and editing (equal).

## Supporting information

Supplementary Material

## Data Availability

The data that support the findings of this study are available from the corresponding author upon reasonable request.
