# Supplementary material for: Microwave‐Assisted Synthesis of Ir—Ni Electrocatalysts for the Oxygen Evolution Reaction in Acidic Electrolyte
Source: ChemistryOpen. 2025 Jul 16;14(11):e202500279. doi: 10.1002/open.202500279 (PMC12598801; doi:10.1002/open.202500279)
Supplement: Supplementary file 1 — Supplementary Material [file OPEN-14-e202500279-s001.pdf]

Supporting information

Microwave-Assisted Synthesis of Ir-Ni Electrocatalysts for the Oxygen Eevolution Reaction in Acidic Electrolyte

Anna Giulia Cardone[a,b], Mattia Bartoli[a], Adriano Sacco[a], Candido Fabrizio Pirri[a,b], Marco Etzi[a,*]

[a] *Center for Sustainable Future Technologies, Istituto Italiano di Tecnologia, Via Livorno 60, 10144 Torino, Italy*

[b] *Department of Applied Science and Technology, Politecnico di Torino, Corso Duca degli Abruzzi 24, 10129 Torino, Italy*

* Corresponding author: marco.etzi@iit.it

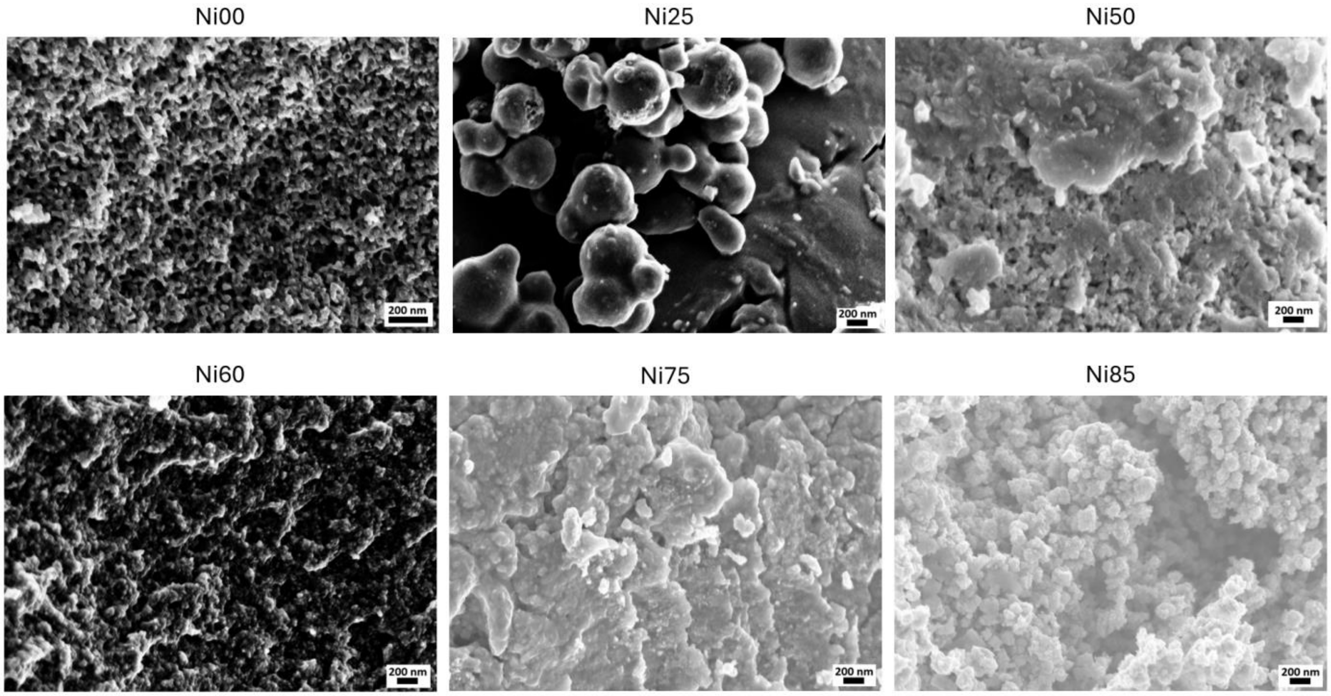

Figure S1: FESEM images of as-prepared powders.

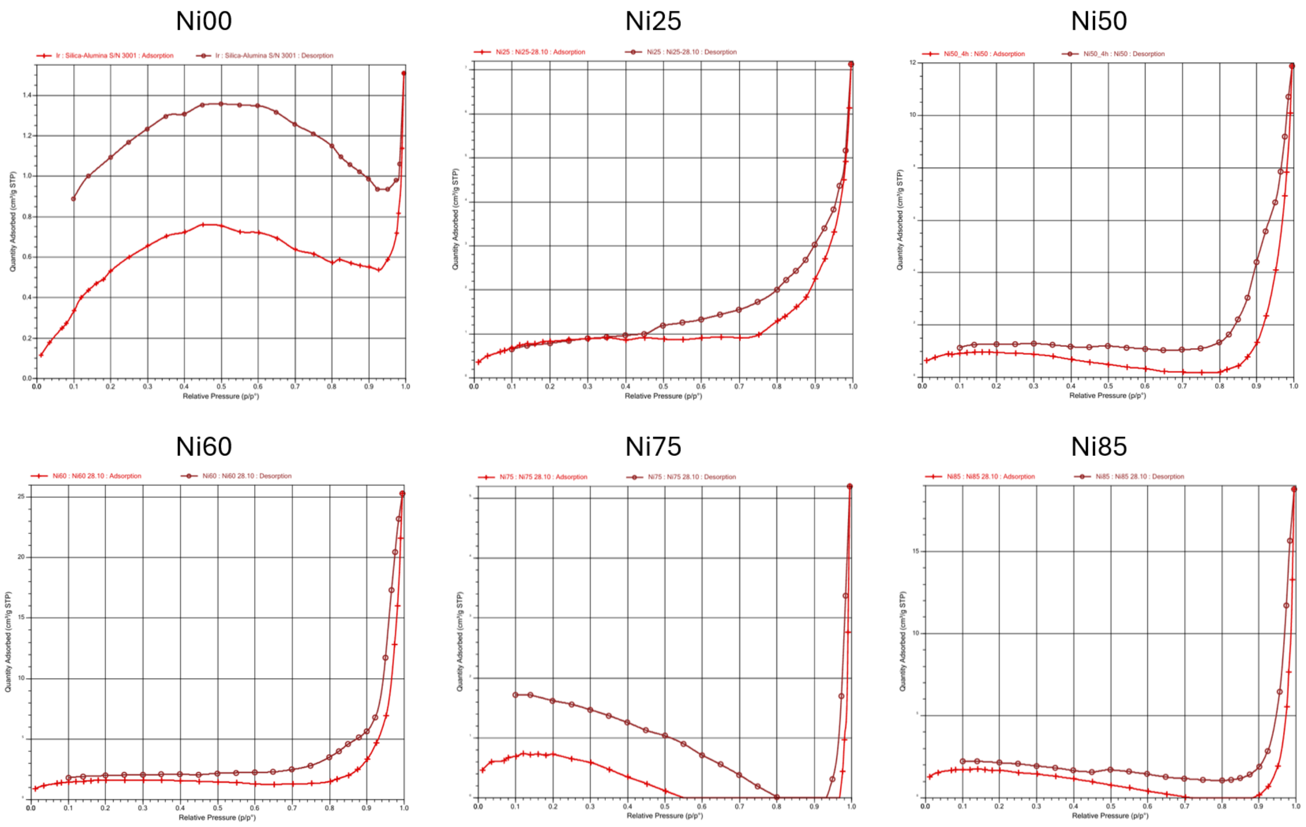

Figure S2: N_2 isotherms of the catalysts.

Table S1: BET results of as-prepared powders.

**Sample**	**Surface area / $m^2\ g^{-1}$**	**Pores volume / $cm^3\ g^{-1}$**	**Average pore size / nm**
Ni00	5.6	0.0017	3
Ni25	4.6	0.0094	14
Ni50	4.4	0.0150	27
Ni60	7.8	0.0320	28
Ni75	3.3	0.0039	11
Ni85	7.3	0.0200	21

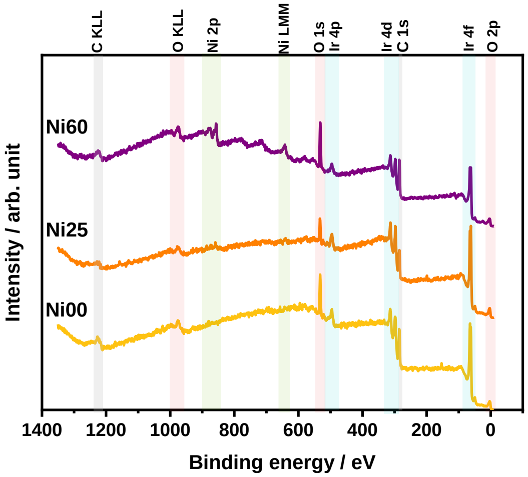

Figure S3. XPS survey spectrum of the Ni00, Ni25 and Ni60 catalysts.

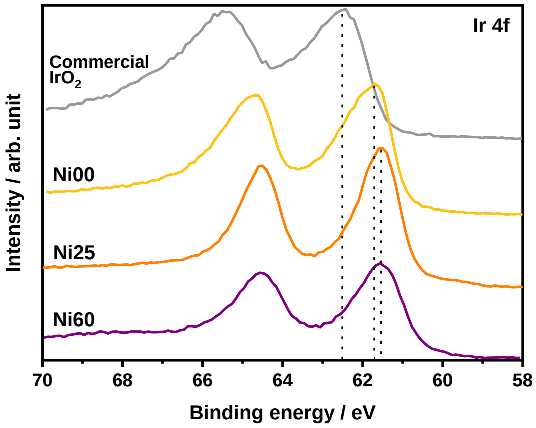

Figure S4. XPS Ir 4f region of Ni00, Ni25, Ni60 and commercial IrO_2 catalyst.

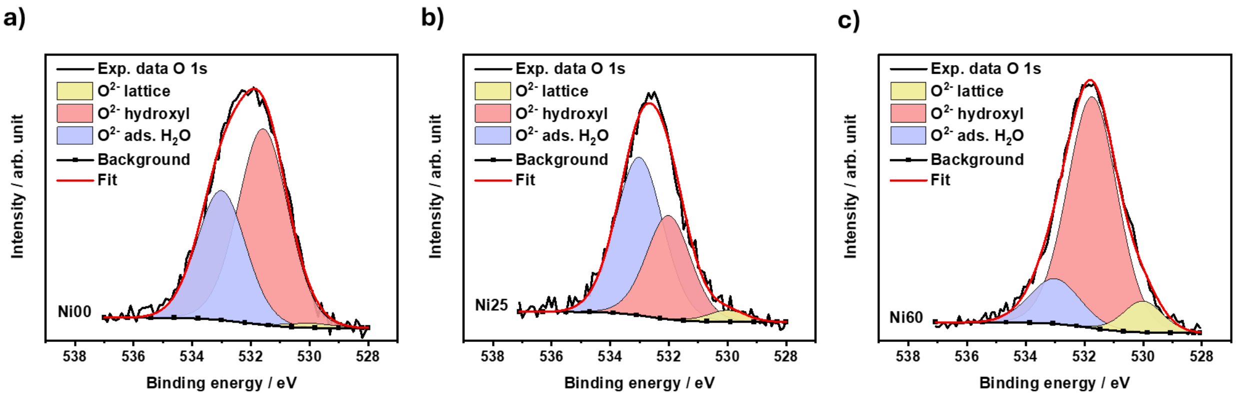

Figure S5. Fitting of the O 1s region of a) Ni00, b) Ni25 and c) Ni60.

Table S2: XPS elemental composition analysis of Ni25 and Ni60 catalysts.

**Sample**	**XPS**	
	**Ni : (Ni + Ir) at %**	**Ir : (Ni + Ir) at %**
Ni25	8	92
Ni60	35	65

Table S3. EDX elemental composition analysis of the spent electrodes after 6-h CA.

Sample	**Ni : (Ni + Ir) / at%**	**Ir : (Ni + Ir) / at%**
Ni25	~ 0	~ 100
Ni50	2 ± 1	98 ± 1
Ni60	3 ± 1	97 ± 1
Ni75	~ 0	~ 100
Ni85	~ 0	~ 100

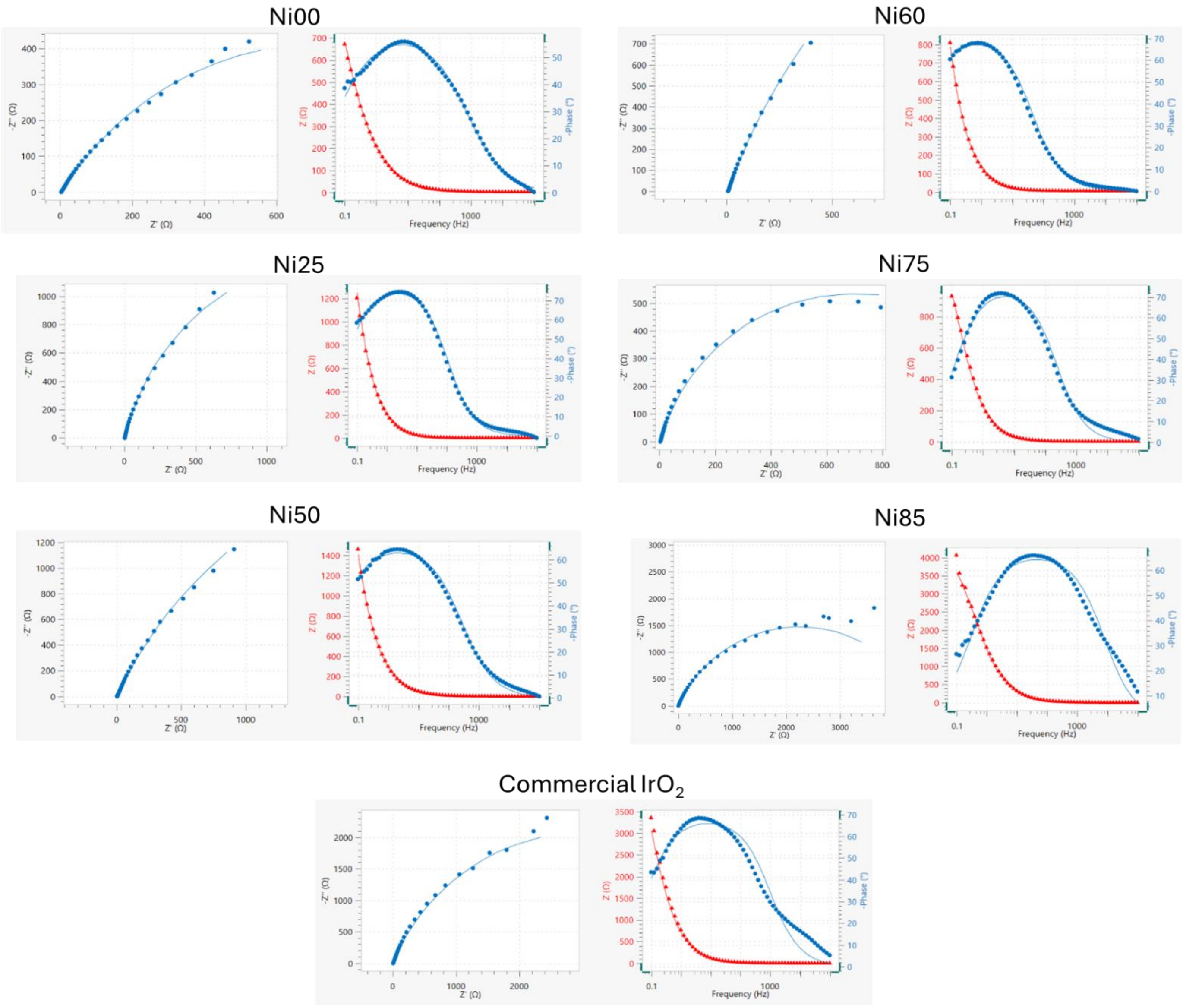

Figure S6. EIS measurements and data fitting to the $R_s(R_{ct}C_{dl})$ equivalent circuit model for the different samples.

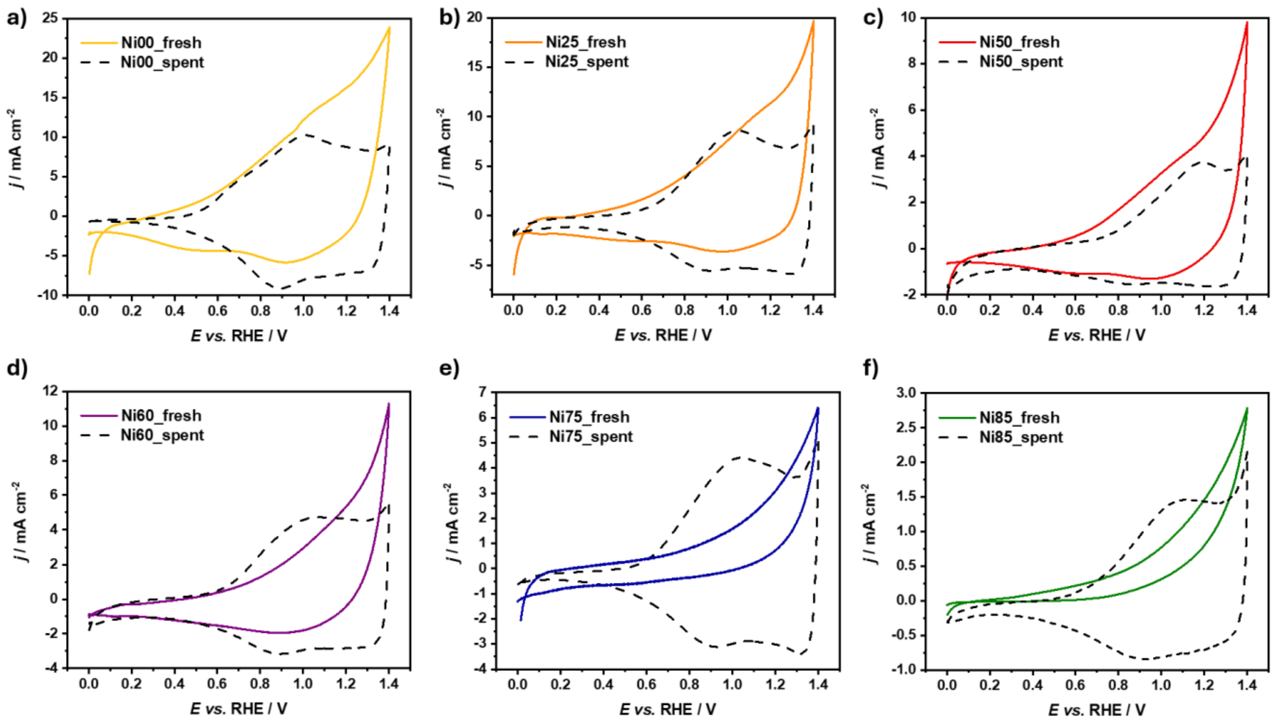

Figure S7. Cyclic Voltammetry of fresh vs. spent electrodes (after a 6-h CA stability test at 1.53 V vs. RHE in 0.5 M H_2SO_4).

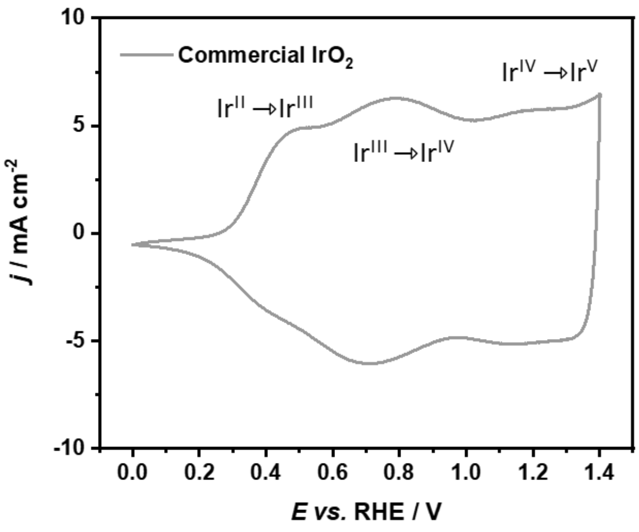

Figure S8. Cyclic Voltammetry of commercial IrO_2 in 0.5 M H_2SO_4.

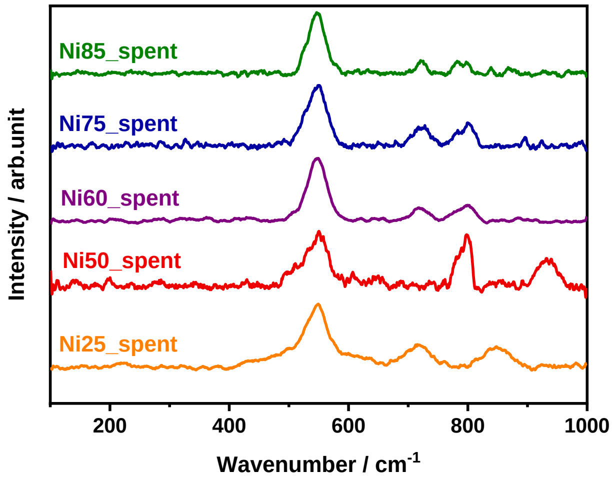

Figure S9. Raman spectra of spent electrodes after 6-h CA.

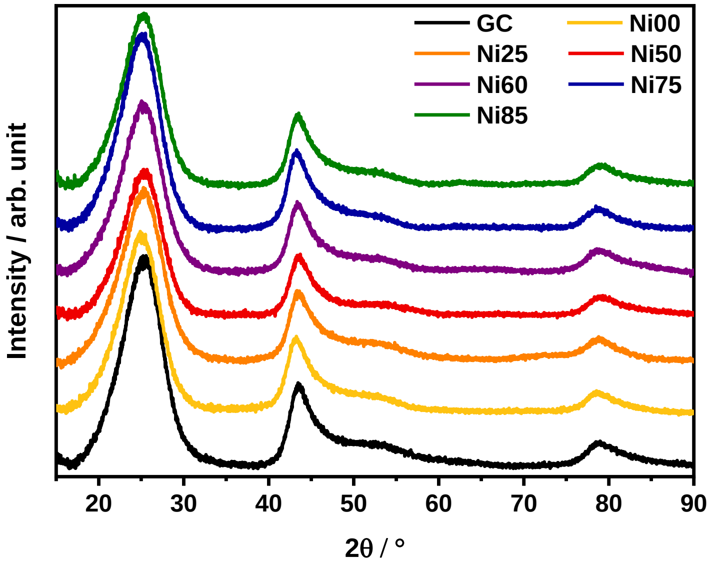

Figure S10. XRD in Bragg-Brentano geometry of the spent electrodes after 6-h CA.

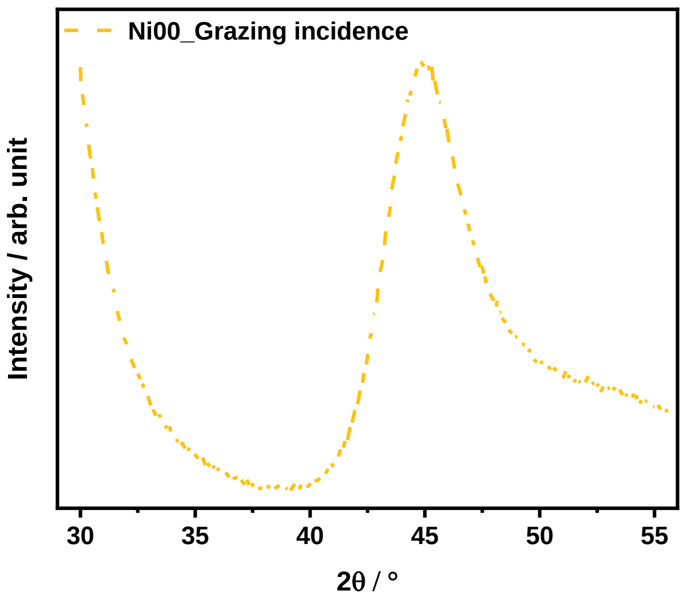

Figure S11. Grazing-incidence XRD of Ni00 spent electrode after 6-h CA.

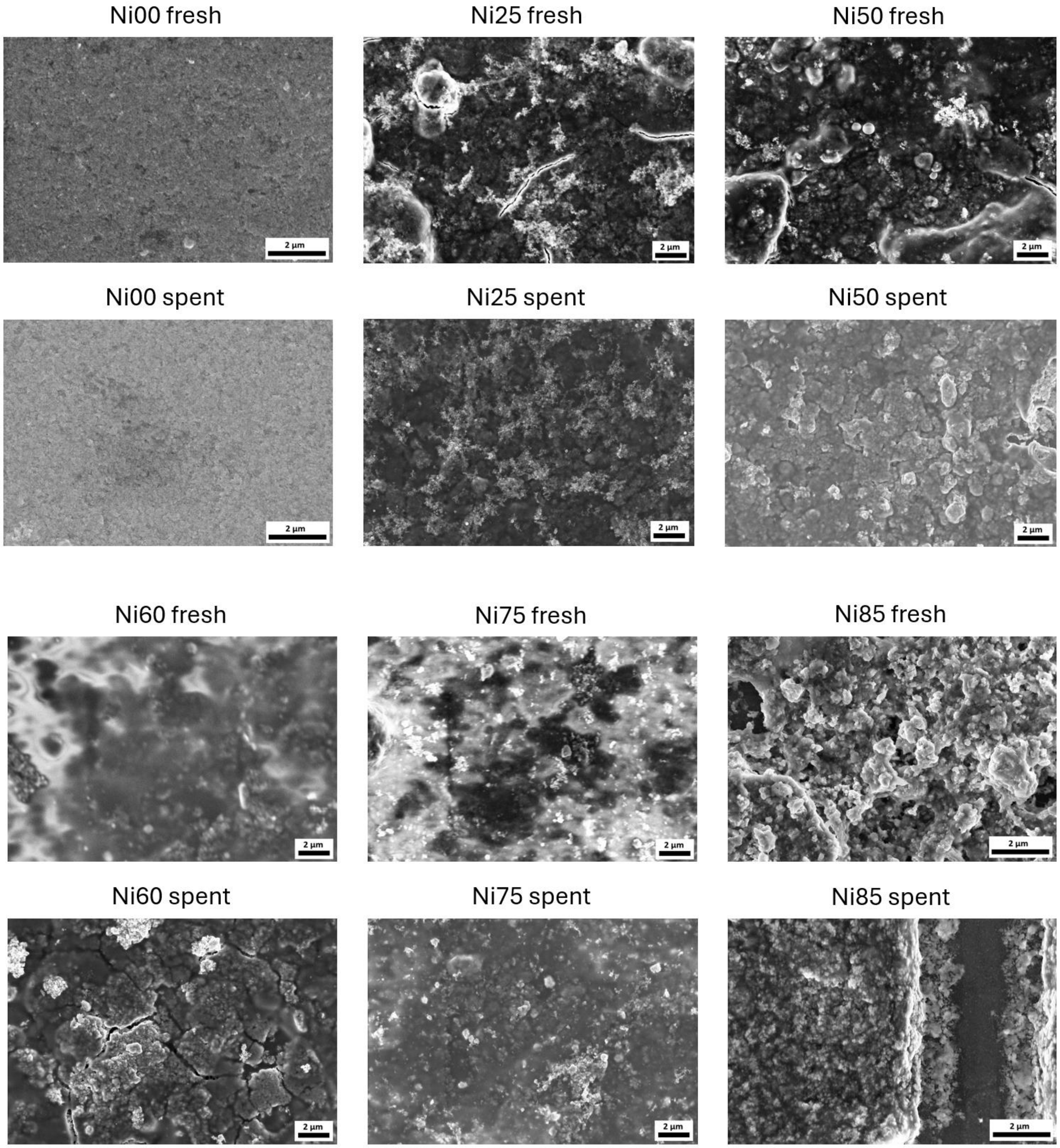

Figure S12. FESEM images of fresh vs. spent electrodes supported on glassy carbon substrates after a 6-h chronoamperometry at 1.53 V vs. RHE in 0.5 M H_2SO_4.
